# *Thunbergia laurifolia* Leaf Extract Inhibits Glutamate-Induced Neurotoxicity and Cell Death through Mitophagy Signaling

**DOI:** 10.3390/antiox10111678

**Published:** 2021-10-25

**Authors:** Wudtipong Vongthip, Chanin Sillapachaiyaporn, Kyu-Won Kim, Monruedee Sukprasansap, Tewin Tencomnao

**Affiliations:** 1 Graduate Program in Clinical Biochemistry and Molecular Medicine, Department of Clinical Chemistry, Faculty of Allied Health Sciences, Chulalongkorn University, Bangkok 10330, Thailand; 6273007137@student.chula.ac.th (W.V.); chanin.sill@gmail.com (C.S.); 2 Research Institute of Pharmaceutical Sciences and College of Pharmacy, Seoul National University, Seoul 151-742, Korea; qwonkim@snu.ac.kr; 3 Food Toxicology Unit, Institute of Nutrition, Mahidol University, Nakhon Pathom 73170, Thailand; 4 Natural Products for Neuroprotection and Anti-Ageing Research Unit, Chulalongkorn University, Bangkok 10330, Thailand; 5 Department of Clinical Chemistry, Faculty of Allied Health Sciences, Chulalongkorn University, Bangkok 10330, Thailand

**Keywords:** autophagy, glutamate, mitophagy, neurodegenerative diseases, oxidative stress, *Thunbergia laurifolia*

## Abstract

Oxidative stress plays a crucial role in neurodegeneration. Therefore, reducing oxidative stress in the brain is an important strategy to prevent neurodegenerative disorders. *Thunbergia laurifolia* (Rang-jued) is well known as an herbal tea in Thailand. Here, we aimed to determine the protective effects of *T. laurifolia* leaf extract (TLE) on glutamate-induced oxidative stress toxicity and mitophagy-mediated cell death in mouse hippocampal cells (HT-22). Our results reveal that TLE possesses a high level of bioactive antioxidants by LC–MS technique. We found that the pre-treatment of cells with TLE prevented glutamate-induced neuronal death in a concentration-dependent manner. TLE reduced the intracellular ROS and maintained the mitochondrial membrane potential caused by glutamate. Moreover, TLE upregulated the gene expression of antioxidant enzymes (SOD1, SOD2, CAT, and GPx). Interestingly, glutamate also induced the activation of the mitophagy process. However, TLE could reverse this activity by inhibiting autophagic protein (LC3B-II/LC3B-I) activation and increasing a specific mitochondrial protein (TOM20). Our results suggest that excessive glutamate can cause neuronal death through mitophagy-mediated cell death signaling in HT-22 cells. Our findings indicate that TLE protects cells from neuronal death by stimulating the endogenous antioxidant enzymes and inhibiting glutamate-induced oxidative toxicity via the mitophagy–autophagy pathway. TLE might have potential as an alternative or therapeutic approach in neurodegenerative diseases.

## 1. Introduction

The accumulation of reactive oxygen species (ROS) contributes to the oxidative stress condition due to the imbalance of redox homeostasis. Normally, ROS are generated during the oxidative phosphorylation process, which is neutralized by antioxidant enzymes including superoxide dismutase (SOD), glutathione peroxidase (GPx) and catalase (CAT). However, these antioxidant systems tend to deteriorate with age. Overproduction of ROS or dysfunction of the antioxidant system can contribute to cellular destruction and cell death. ROS are the most common free radicals, which cause damage to the cells, especially neurons due to their high metabolic rate [1,2]. This is one of the critical factors of neurodegenerative diseases. Alzheimer’s disease (AD) is an important disease that accounts for 60–70% of dementia cases [3,4,5], with other diseases such as Parkinson’s disease (PD), Huntington’s disease (HD), multiple sclerosis (MS) and amyotrophic lateral sclerosis (ALS) also contributing significantly towards cognitive decline. Various researchers believe that the inhibition of ROS accumulation with antioxidant compounds or drugs could be effective in treating neurodegenerative diseases [6,7].

Glutamate is a neurotransmitter involved in the excitatory process, and it is significant for brain functions such as memory and learning. However, a high concentration of glutamate can cause damage and death to neuronal cells, leading to various neurodegenerative diseases, including AD [8,9,10]. Previous reports indicate that high glutamate content in the hippocampus is related to the accumulation of amyloid beta and tau proteins [11]. Glutamate can trigger ROS and oxidative-stress-induced neuronal cell death through both glutamate receptor and non-glutamate receptor (cystine/glutamate antiporter) mechanisms. Excessive extracellular glutamate content can interrupt cystine uptake through the cystine/glutamate antiporter system, leading to the depletion of intracellular antioxidants (glutathione) and accumulation of ROS [9,10]. The consequences of this process cause oxidative damage in neuronal cells, especially mitochondria, the first damaged organelles, which are then cleared and recycled [12].

Autophagy is a mechanism that involves the clearance and recycling of damaged and unnecessary components in the cells. The damaged and unnecessary components are enveloped with a double-membrane vesicle “Autophagosome”, then fused with lysosome to be degraded and recycled [13,14,15]. However, the continuous trigger of autophagy upon high ROS production results in an inappropriate autophagic process, reaching the point of no return and causing autophagic cell death. Furthermore, one type of selective autophagy is mitophagy, which is involved in the programmed mitochondrial elimination mechanism, and maintains a balance between mitochondrial quantity and quality [16,17]. This process can be initiated in the condition of prolonged ROS, hypoxia, starvation, and cell senescence [18], leading to mitochondrial membrane depolarization or a loss of mitochondrial membrane potential status. This process is also a significant regulator of other types of cell death, such as apoptosis and necrosis in the nervous system. Supporting evidence suggests that mitophagy and autophagy are related to oxidative stress conditions and cell death in neurons, causing neurodegenerative disease [19,20]. 

*Thunbergia laurifolia* (Rang jued) belongs to the Acanthaceae family. Rang jued is a well-known herbal tea in Thailand with anti-inflammatory, anti-bacterial and antioxidant properties. Raw leaf of Rang jued is widely used as a detoxification agent against pesticides [21]. However, the neuroprotective effects of Rang jued leaves on neuronal cells have not yet been studied. Therefore, the present study attempted to investigate, for the first time, the neuroprotective effects of *T. laurifolia* leaf extract (TLE) against glutamate-induced oxidative stress and neuronal cell death through autophagy and mitophagy processes and to further elucidate its underlying mechanisms against oxidative glutamate toxicity using a mouse hippocampal neuronal cell line (HT-22) as a neurodegenerative cellular model.

## 2. Materials and Methods

### 2.1. Chemicals and Reagents 

The analytical-grade reagents used in the extraction process were purchased from RCI Labscan (Bangkok, Thailand). The 2,7-dihydrofluorescein diacetate (H_2_DCF-DA) was obtained from Thermo Scientific (Waltham, MA, USA). Dulbecco’s modified Eagle medium (DMEM), sodium selenite, chloroquine, 4′,6-diamidino-2-phenylindole (DAPI) and L-glutamic acid were purchased from Sigma-Aldrich (St. Louis, MO, USA). The 3-(4,5-dimethylthiazol-2-yl)-2,5-diphenyl tetrazolium bromide (MTT) was obtained from Biobasic (Markham, ON, Canada). CytoTox 96 ^®^ Non-Radioactive Cytotoxicity assay was purchased from Promega (Madison, WI, USA). Alexa Fluor 488, carbonyl cyanide m-chlorophenylhydrazone (CCCP), mouse monoclonal anti-β-actin antibody, mouse monoclonal anti-rabbit IgG, HRP-linked antibody, rabbit polyclonal anti-LC3B antibody, rabbit monoclonal anti-TOM20 (D8T4N) antibody and tetramethylrhodamine ethyl ester (TMRE) were purchased from Cell Signaling Technology (Denvers, MA, USA). All primers, the AccuPower^®^ RT Premix kit and AccuPower^®^ 2X GreenStar^TM^ qPCR Master Mix were purchased from Bioneer (Daejeon, South Korea). Mitotracker Red CMXRos was obtained from Molecular Probes (Eugene, OR, USA).

### 2.2. Plant Material and Extraction 

*T. laurifolia* leaves were collected from HRH Princess Maha Chakri Sirindhorn Herbal Garden in Rayong Province, Thailand. The plant was identified at the herbarium of Kasin Suvatabhandhu (Department of Botany, Faculty of Science, Chulalongkorn University, Thailand), with the voucher specimen A013700 (BCU). Plant materials were washed 3 times and dried in a ventilated incubator at 40 °C. The dried leaves were mashed into powder. Forty grams of TLE powder was packed in the cellulose extraction thimble and extracted in Soxhlet apparatus. Then, 400 mL of extracting solvent (ethanol) was added to the boiling flask. After 36 h of extraction, the extracted solvent was evaporated and concentrated with a rotary evaporator and miVac concentrator, respectively. The yield of ethanol extract was 7.96%.

### 2.3. Qualitative Bioactive Compounds of TLE by LC–MS Analysis

The phytochemical profiling of TLE was analyzed by liquid chromatography–mass spectrometry (LC–MS), with a DionexTM Ultimate 3000 UHPLC system (Thermo Fisher Scientific, Rockford, IL, USA) coupled with a high-resolution micrOTOF-Q III (Bruker Daltonics, Bremen, Germany) at the Institute of Systems Biology (Universiti Kebangsaan Malaysia, Malaysia). The AcclaimTM Polar Advantage II C18 column (3 mm × 150 mm, 3 μm particle size) (Thermo Fisher Scientific, Rockford, IL, USA) was used as the chromatographic column with a flow rate of 400 μL/min. The gradient elution conditions were as follows: 5% B (0–3 min); 80% B (3–10 min); 80% B (10–15 min) and 5% B (15–22 min), where solvent A is 0.1% formic acid in water and solvent B is 100% acetonitrile. The mass analysis was detected by electrospray ionization (ESI) with ion positive mode. Then, the m/z values from the experiment were compared with the METLIN (La Jolla, CA, USA) and the KNApSAcK (Keyword Search Web Version 1.000.01) databases, with the acceptance of mass error less than 30 parts per million (ppm). 

### 2.4. Cell Culture 

The mouse hippocampal neuronal HT-22 cells were a generous gift from Prof. David Schubert (The Salk Institute, San Diego, CA, USA). HT-22 cells were cultured with Dulbecco’s modified Eagle’s medium (DMEM) containing 10% fetal bovine serum, 100 U/mL penicillin, and 100 µg/mL streptomycin. Cells were incubated under 5% CO_2_ in a humidified atmosphere at 37 °C. The culture medium was renewed every 3 days and cells were grown to 80–85% confluence for the experiments. The passage number of HT-22 cells was selected for use in the range No. 9–20, throughout the experiments.

### 2.5. Cell Viability Assay

The cell viability of mouse hippocampal cells was assessed by 3-(4,5-Dimethylthiazolyl)-2,5-diphenyltetrazolium bromide (MTT) assay. HT-22 cells were seeded in 96-well plates at a density of 3000 cells per well for 18–24 h. Cells were pre-treated with different concentrations of TLE for 24 h, followed by glutamate (5 mM) in complete medium at 37 °C in a 5% CO_2_ incubator with a humidified atmosphere for 18 h. Then, 20 µl of 5 mg/mL MTT cell viability reagent (Biobasic, Markham, ON, Canada) was added for 3 h. The supernatant was removed and the formazan crystals were dissolved with 150 µl dimethyl sulfoxide (DMSO), and the absorbance was measured using a microplate reader (Enspire, Perkin-elmer, Waltham, MA, USA) at 550 nm. The percentages of cell viability were calculated and compared with the control.

### 2.6. Cytotoxicity Assay

Cytotoxicity was measured from LDH enzymes released from damaged cells. HT-22 cells were seeded in 96-well plates at a density of 3000 cells per well overnight. Cells were pre-treated with different concentrations of TLE for 24 h, followed by glutamate (5 mM) in complete medium at 37 °C in a 5% CO_2_ incubator with a humidified atmosphere for 18 h. After the incubation period, 50 µL of supernatant was pipetted and added to a new 96-well plate. Cell cytotoxicity was analyzed by CytoTox 96^®^ Non-Radioactive Cytotoxicity Assay (Promega, Madison, WI, USA) according to the manufacturer’s instructions. Lysis buffer was added for 30 min and was used as a cell lysis control. To measure the LDH enzyme, 50 µl of cytotox reagent was added in to the new 96-well plate from the previous description and incubated for 30 min at room temperature. At the end of the incubation period, 50 µl of stop solution was added to stop the reaction. After that, the absorbance was measured using a microplate reader (Enspire, Perkin-Elmer, Waltham, MA, USA) at 490 nm. The percentages of LDH release were calculated and compared with the control.

### 2.7. Intracellular ROS Assay

Intracellular ROS was measured using H_2_DCF-DA. Shortly, HT-22 cells were seeded in 12-well plates at a density of 10,000 cells per well. Cells were pre-treated with selected concentrations of TLE for 24 h followed by glutamate (5mM) in complete medium at 37 °C in a 5% CO_2_ incubator with a humidified atmosphere for 18 h. After the incubation period, 10 µm H_2_DCF-DA was added and incubated at 37 °C for 45 min. The stained cells were washed twice with cold phosphate-buffered saline (PBS) before trypsinization and resuspended in cold PBS. The dihydroethidium (DHE) fluorescence was analyzed with a flow cytometer (FACSCalibur, BD biosciences, San Jose, CA, USA) at excitation wavelength 488 nm and emission wavelength 525 nm.

### 2.8. Mitochondrial Membrane Potential Staining (TMRE) Assay

The mitochondrial membrane potential was measured by staining with the tetramethylrhodamine ethyl ester (TMRE). HT-22 cells were grown on a cover slip for 18–24 h and pre-treated with selected concentrations of TLE for 24 h, followed by glutamate (5 mM) in the complete medium at 37 °C in a 5% CO_2_ incubator with a humidified atmosphere for 12 h. After the incubation period, the cells were stained with 200 nM TMRE solution for 30 min. The mitochondrial membrane potential disruption agent carbonyl cyanide 3-chlorophenylhydrazone (CCCP) (50 µM) was used as a positive control. After washing with PBS, cells were mounted and analyzed with a confocal laser scanning microscope (LSM 700) (Carl Zeiss, Jena, Germany) at excitation wavelength 550 nm and emission wavelength 580 nm.

### 2.9. Western Blot Analysis

HT-22 cells were seeded in 6-well plates for 18-24 h. Afterwards, HT-22 cells were treated with selected concentrations of TLE (24 h) followed by glutamate (5 mM) in complete medium at 37 °C in a 5% CO_2_ incubator with a humidified atmosphere for 18 h before harvest. The collected cells were washed with cold PBS and lysed on ice in pre-cooled RIPA lysis buffer containing protease inhibitors. A total of 20 µg of proteins was added in each lane of 12% acrylamide gel (Biorad, Hercules, CA, USA). After the separation step, the proteins were transferred to PVDF membranes (GE Healthcare) and blocked with 5% nonfat dry milk (Biorad, Hercules, CA, USA) in Tris-buffered saline and 0.1% Tween 20 detergent (1XTBS-T) for 1 h. Target proteins were probed with primary antibodies against LC3B (1:8000, 2775, Cell Signaling Technology, USA), TOM20-D8T4N (1:10000, 42406, Cell Signaling Technology, USA) and β-actin (1:16000, Cell Signaling Technology, Danvers, MA, USA) at 4 °C overnight. Then, the proteins were probed with a HRP conjugated secondary antibody (1:16000, Cell Signaling Technology, Danvers, MA, USA) at room temperature for 1 h. Finally, the proteins were detected using an enhanced chemiluminescence (ECL) Western Blot kit. The proteins were analyzed using NIH image J. 

### 2.10. Immunofluorescent Colocalization Analysis

HT-22 cells were seeded on coverslips in 6-well plates. The cells were treated with TLE (24 h) followed by glutamate (5 mM) in complete medium at 37 °C in a 5% CO_2_ incubator with a humidified atmosphere for 18 h before harvest. After washing with PBS, cells were stained with Mitotracker (200 nM) for 30 min and fixed with 4% paraformaldehyde at room temperature for 15 min then permeabilized in 0.3% Triton X-100 in PBS for 10 min and blocked with 2% FBS for 1 hr. Then, cells were probed with monoclonal rabbit antibody to LC3B (1:200 dilution) at 4 °C overnight. After washing with PBS, cells were probed with Alexa 488 anti-rabbit secondary antibody (Cell Signaling Technology) at room temperature for 1 h. Then, cells were washed with PBS and stained with DAPI. After mounting with prolonged anti-fade, slides were analyzed with a confocal laser scanning microscope (LSM 700) (Carl Zeiss, Jena, Germany).

### 2.11. Real-Time PCR Analysis

HT-22 cells were seeded in 6-well plates. The cells were treated with TLE for 12 h before harvest. Total RNA was extracted by Trizol reagent and RNA concentration was measured using a Nanodrop spectrometer (Thermo Scientific, Rockford, IL, USA). RNA was converted to complementary DNA (cDNA) by reverse transcription using AccuPower RT Premix kit (Bioneer, South Korea). The cDNA was used as a sample for the real-time PCR step. Real-time PCR assay was performed using AccuPower 2X GreenStar^TM^ qPCR Master Mix (Bioneer, South Korea) in Exicycler^TM^ 96 (Bioneer, Daejeon, South Korea) using gene-specific primers (Table 1) [22,23]. The mRNA expression level was calculated by the delta-delta Ct method with β-actin as an internal control. 

### 2.12. Molecular Docking

Co-crystal structures of Kelch-like ECH-associated protein 1 (KEAP1) complexed with 2-[[4-[2-hydroxy-2-oxoethyl-(4-methoxyphenyl)sulfonyl-amino]-3-phenylmethoxy-phenyl]-(4-methoxyphenyl)sulfonyl-amino]ethanoic acid (GX8) (PDB ID: 6HWS, https://www.rcsb.org/structure/6HWS, accessed on 23 August 2021) and PTEN-induced kinase 1 (PINK1) complexed with ubiquitin (PDB ID: 6EQI, https://www.rcsb.org/structure/6EQI, accessed on 23 August 2021) [24], and E3 ubiquitin–protein ligase parkin (PDB ID: 5C1Z, https://www.rcsb.org/structure/5C1Z, accessed on 23 August 2021) [25] were obtained from RCSB Protein Data Bank. Protein and compound files were prepared following the previous procedure [26]. Briefly, the proteins were prepared by removing all waters and original ligands, as well as adding missing hydrogen atoms and Kollman charges using the AutoDockTools-1.5.6 program. The protein files were saved in PDBQT format for further analysis. Ligand structures were drawn by BIOVIA Draw 2019 software. The clean geometry of all ligands was performed by using BIOVIA Discovery Studio 2020, then the ligand structures were saved in PDB file. After that, the format files were converted to PDBQT format by using AutoDockTools-1.5.6.

Molecular docking was performed by using AutoDock Vina software [27] with all default parameters, following the procedure of Forli S et al. [28]. For KEAP1, the grid box was set based on the original inhibitor with the number of points in the xyz dimension of 30 × 30 × 30, spacing 0.375 Å and center grid box at -13.549 × 6.01 × 13.387 (xyz). The gid box of PINK1 docking was set to 40 × 40 × 40 points in the xyz dimension, spacing 0.375 Å and center grid box at 62.7479 × 5.4715 × 11.7265 (xyz) [29]. At the catalytic domain of parkin, the grid box was adjusted to 35 × 35 × 35 xyz dimension points, spacing 0.375 Å and center grid box at -12.543 × 34.705 × 27.038 (xyz). The best conformation exhibited the lowest binding energy. The protein–ligand interaction was visualized by BIOVIA Discovery Studio 2020.

### 2.13. Lipinski’s Rule of Five Parameters and ADMET Property Analysis

Physicochemical properties of all compounds were predicted using the SwissADME online database (http://www.swissadme.ch accessed on 1 October 2021) [30]. The drug-likeness of all compounds was considered by Lipinski’s rule of five parameters: molecular weight ≤ 500; the number of hydrogen bond acceptors ≤ 10; the number of hydrogen bond acceptors ≤ 5; and MlogP ≤ 4.15 [31]. Moreover, the pharmacokinetic properties, adsorption, distribution, metabolism, excretion, and toxicity (ADMET), of all compounds were predicted by using the pkCSM online database (http://biosig.unimelb.edu.au/pkcsm/prediction accessed on 1 October 2021) [32].

### 2.14. Statistical Analysis

All the results are presented as the mean ± standard error of the mean (SEM) from at least three independent experiments and were analyzed by SPSS 16.0 software. One-way ANOVA was used for the evaluation of statistical significance with a post hoc Dunnett’s test and Bonferroni. A *p*-value of less than 0.05 was considered statistically significant.

## 3. Results

### 3.1. Characterization of Bioactive Compounds from TLE 

The putative compounds of TLE were determined by LC–MS analysis. The chromatographic peaks were identified by comparing the m/z value with the MS databases in ion positive mode, as shown in the supplementary data (Figure S1). Epicatechin (6.08%), apigenin-7-*O*-glucoside (5.14%), 7-hydroxycoumarin (4.29%), apiin (3.85%) and betaine (2.00%) were found to be the five major bioactive compounds in TLE by LC–MS.

### 3.2. TLE Attenuates Glutamate-Induced Toxicity in HT-22 Cells

**Figure 1:**
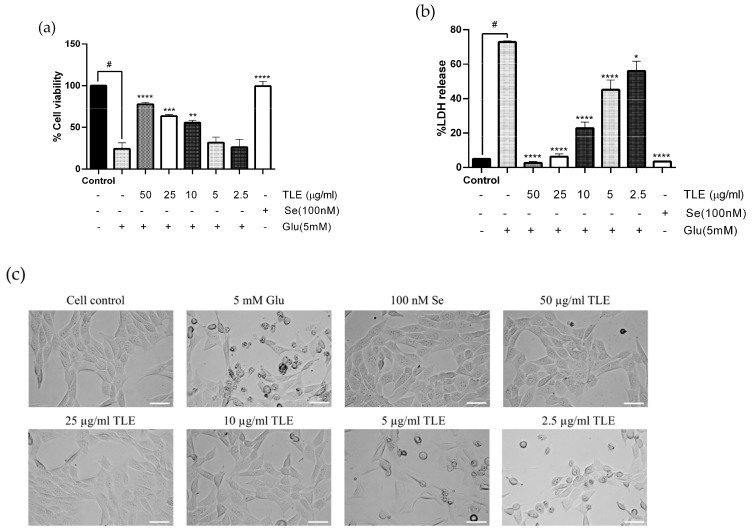
TLE attenuates glutamate-induced toxicity in HT-22 cells. The effect of TLE on glutamate-induced cytotoxicity in HT-22 was assessed by MTT assay and LDH assay. HT-22 cells (passage 12–15) were pre-treated with TLE at different concentrations (2.5–50 µg/mL) and selenium as a positive control for 24 h, followed by 5 mM glutamate for 18 h. Bar graphs show the % cell viability (**a**) and % LDH release (**b**). The morphology of HT-22 cells was visualized under the inverted light microscope (scale bar is 50 µm) (**c**). The data were collected from at least three independent experiments and the results are shown as mean ± SEM. * *p* value < 0.05, ** *p* value < 0.01, *** *p* value < 0.005, **** *p* value < 0.001 compared with glutamate treatment group, ^#^ *p* value < 0.001 compared with untreated control.

To evaluate the effect of TLE on glutamate-induced oxidative toxicity in neurons, we used the HT-22 mouse hippocampal cell line as a model. From our previous study, a toxic level of glutamate (5 mM) caused HT-22 cell death after 18 h of treatment [22,23]. TLE (2.5–50 µg/mL) did not show any toxic effect in HT-22 cells [33]; therefore, TLE at 2.5-50 µg/mL was used in this experiment. In the present study, the HT-22 cells were pre-treated with TLE at various doses, followed by glutamate (5 mM). According to Kumari and Mehta et al. (2012), selenium (in the form of sodium selenite), which exerted a neuroprotective effect against glutamate toxicity, was used as a positive control [34]. The results show that 5 mM of glutamate caused about 70% cell death in HT-22 cells. However, TLE at 12.5-50 µg/mL significantly increased cell viability in a dose-dependent manner (*p* < 0.001). TLE at 50 µg/mL increased cell viability to approximately 90%, similar to that of 100 nM selenium (positive control), as shown in Figure 1a. Moreover, the cell cytotoxicity (LDH) assay was used to support cell viability. The cell cytotoxicity results show that TLE at 2.5-50 µg/mL and selenium (100 nM) reduced the toxicity of glutamate in a dose-dependent manner (Figure 1b). Furthermore, the cell morphology examination under the light microscope showed that glutamate caused nuclear condensation and cell shrinkage, while pre-treatment of cells with TLE and selenium sustained the cell morphology (Figure 1c), with 50 µg/mL of TLE being the most effective concentration to reduce cytotoxicity from glutamate. Thus, the results show that TLE exerts a neuroprotective effect against glutamate-induced toxicity. 

### 3.3. TLE Inhibits Glutamate-Induced Intracellular ROS Generation 

**Figure 2:**
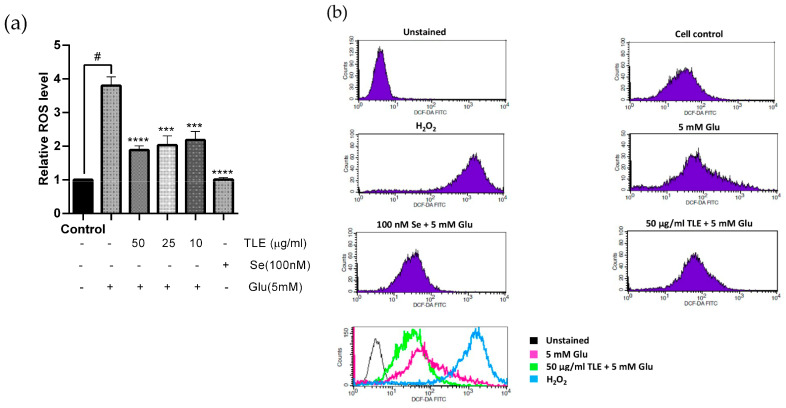
TLE inhibits glutamate-induced intracellular ROS production. Flow cytometry was used to detect the fluorescence intensity of DCF. HT-22 cells (passage 15,16,18,19) were pre-treated with TLE at different concentrations (10–50 µg/mL) or selenium (100 nM) for 24 h and then exposed to 5 mM glutamate for 18 h. (**a**) The bar graph of each treatment shows the relative ROS level in HT-22 cells. (**b**) The flow cytometry histogram and the overlay of the histogram of each treatment; unstained (black), TLE treatment with glutamate (green), 5 mM glutamate treatment (pink) and 250 mM H_2_O_2_ treatment (blue). The data were collected from at least three independent experiments and the results are shown as mean ± SEM. *** *p* value < 0.005, **** *p* value < 0.001 compared with glutamate treatment group, ^#^ *p* value < 0.001 compared with untreated control.

Glutamate causes cytotoxicity in the neuronal cells by inducing the production of ROS. To determine the effect of TLE against glutamate-induced oxidative stress, the intracellular ROS was analyzed from the fluorescent intensity using H_2_DCF-DA probe. HT-22 cells were pre-treated with TLE or selenium, which protected HT-22 cells from glutamate toxicity in previous experiments, followed by 5 mM glutamate for 18 h. The results show that glutamate notably increased the intracellular ROS to 4 times that of the non-treatment group. However, TLE and selenium significantly inhibited ROS production in HT-22 cells in a dose-dependent manner when compared with the glutamate treatment group (Figure 2a). Flow cytometry histograms of each treatment are shown in Figure 2b. Upon glutamate treatment, the histogram was found to shift to the right, which shows the increasing H_2_DCF-DA intensity (increase in intracellular ROS) compared with the ROS control (250 mM H_2_O_2_). However, pre-treatment with TLE can reduce the intracellular ROS, similar to the untreated control (no shift). Thus, TLE at 50 µg/mL is the most effective dose to prevent glutamate-induced intracellular ROS in HT-22 cells.

### 3.4. TLE Sustains the Membrane Potential of Mitochondria

**Figure 3:**
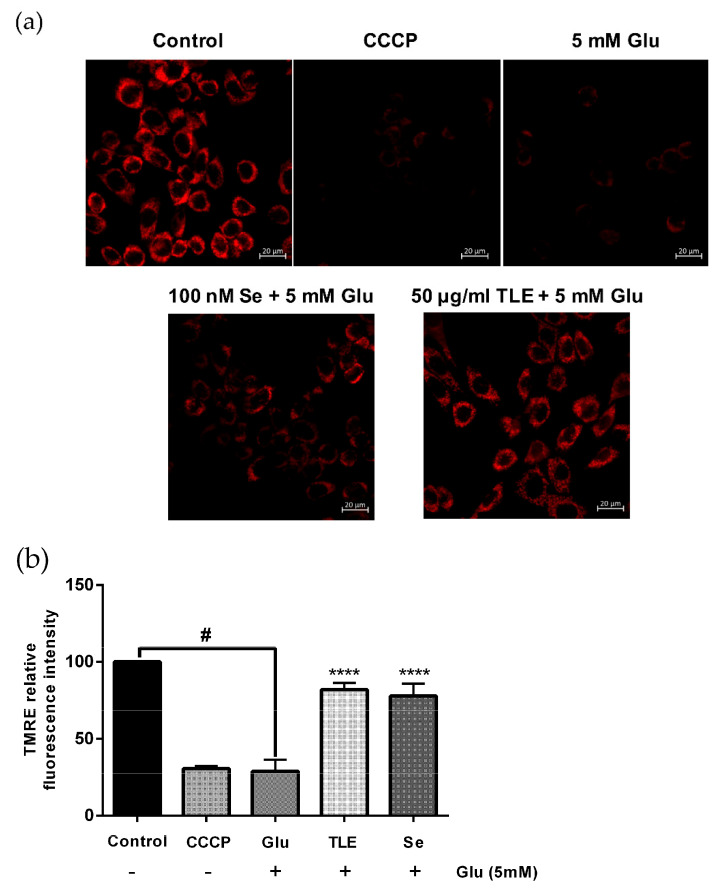
TLE restores mitochondrial membrane potential status. HT-22 cells (passage 911) were pre-treated with TLE at 50 µg/mL or selenium for 24 h followed by 5 mM glutamate. (**a**) The status of the mitochondrial membrane potential of each treatment was stained using a TMRE probe. They were observed under the confocal laser scanning microscope; scale bar 20 µm. (**b**) Data are expressed as the relative TMRE level of the non-treated control. CCCP, mitochondrial uncoupling agent. Values were collected from at least three independent experiments and the results are shown as mean ± SEM (*n* = 3). **** *p* value < 0.001 compared with glutamate treatment group, ^#^ *p* value < 0.001 compared with untreated control.

Mitochondrial membrane potential is sensitive to oxidative stress, resulting in the loss of membrane potential and then neuronal cell death. In order to investigate the membrane potential status of mitochondria, HT-22 cells were stained with TMRE (a fluorescent dye that stains active mitochondria). As TLE (50 µg/mL) showed effective reduction of intracellular ROS generation, this dose was selected for this experiment. Figure 3a shows that the control group (untreated group) emitted a high fluorescence intensity, while the glutamate treatment group caused the loss of fluorescence intensity, which was related to the loss of mitochondrial membrane potential. Quantification of the relative fluorescence intensity of TMRE exhibited that glutamate significantly decreased the fluorescence intensity compared with the control group (Figure 3b). The mitochondrial uncoupling agent (CCCP), which was used as the system control of this experiment, also showed a loss of fluorescence intensity similar to the glutamate treatment group. CCCP interferes with the proton gradient and disrupts the membrane potential of mitochondria. Interestingly, the pretreated cells with TLE and selenium (positive control) prior to glutamate treatment could sustain the mitochondrial membrane potential compared with the glutamate treatment group. These results suggest that TLE rescue the loss of mitochondrial membrane potential in response to glutamate.

### 3.5. TLE Upregulates the mRNA Expression Level of Antioxidant Enzyme Genes

**Figure 4:**
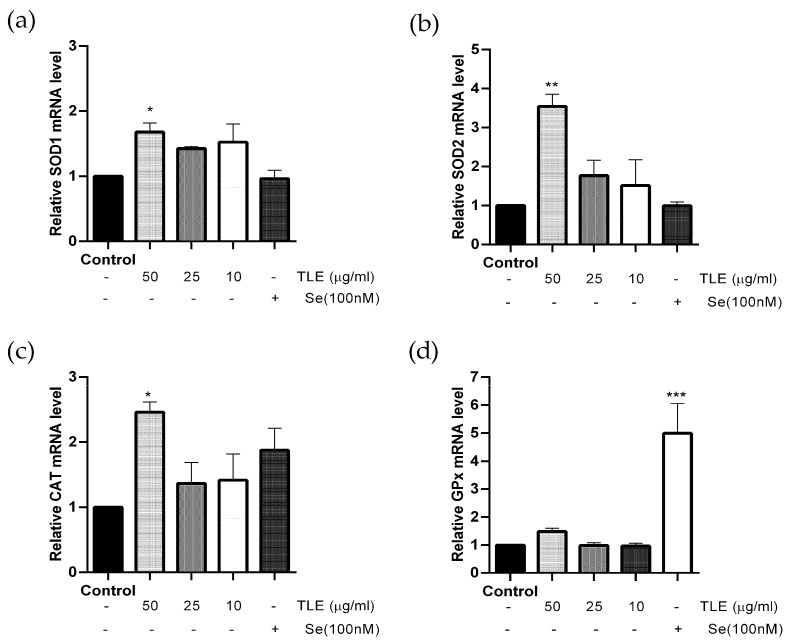
TLE increases the mRNA expression of antioxidant enzyme genes. HT-22 cells (passage 13,15,16) were treated with TLE (10-50 µg/mL) for 12 h and then analyzed with the real-time PCR technique. The mRNA expression levels of (**a**) SOD1, (**b**) SOD2, (**c**) CAT and (**d**) GPx were normalized with β-actin and the results are shown as fold change in mRNA expression with mean ± SEM (*n* = 3). * *p* value < 0.05, ** *p* value < 0.01, *** *p* value < 0.005 compared with untreated control.

Antioxidant enzymes are an important defense mechanism against ROS in the cells, and play a significant role in neutralizing, stabilizing and deactivating free radicals [1,35]. In this study, HT-22 cells were treated with TLE at different concentrations (10–50 µg/mL) and the mRNA expression of SOD1, SOD2, GPx and CAT enzymes were analyzed with real-time PCR. The results indicate that the highest concentration (50 µg/mL) of TLE significantly stimulated the mRNA level of SOD1, SOD2 and CAT (Figure 4a–c), while the mRNA level of GPx was only activated by selenium (Figure 4d) compared to the untreated control. Thus, this result suggests that TLE can upregulate the endogenous antioxidant enzyme genes in HT-22 cells.

**Figure 5:**
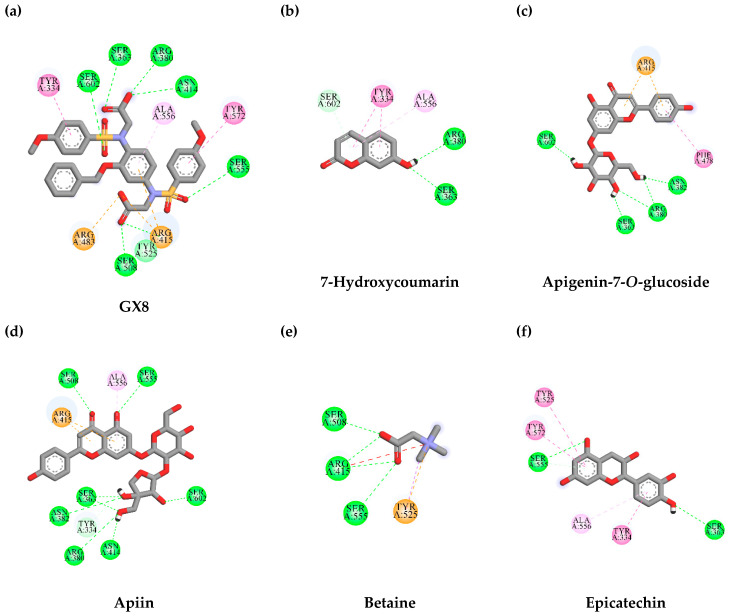
Schematics of amino acid interactions of KEAP1 and candidate ligands: (**a**) GX8, (**b**) 7-hydroxycoumarin, (**c**) apigenin-7-*O*-glucoside, (**d**) apiin, (**e**) betaine and (**f**) epicatechin. Green, pink and orange dashes indicate hydrogen, hydrophobic and electrostatic bonds, respectively.

In order to clarify and explain the interaction between identified compounds from TLE and the binding site of KEAP1, a negative regulator of Nrf2, molecular docking was studied. The KEAP1 in complex with GX8 (PDB ID: 6HWS) was retrieved from the RCSB Protein Data Bank. Initially, the GX8 was removed from the complex, then the removed ligand was re-docked into the original binding site of KEAP1 by using AutoDock Vina. The re-docking results show that GX8 was capably docked into the original binding pocket with a binding energy of -8.6 kcal/mol; this value was set as a benchmark value for the result interpretation of the candidate ligands. The ligand provides a binding energy less than the reference value, which is considered a potential KEAP1 inhibitor. Interestingly, apigenin-7-*O*-glucoside was capably docked into the binding site of KEAP1 with a binding energy lower than that found in GX8 (the reference ligand). Interactions between KEAP1 and candidate ligands are presented in Table 2 and Figure 5.

### 3.6. TLE Inhibits Glutamate-Induced Excessive Mitophagy in HT-22 Cells

**Figure 6:**
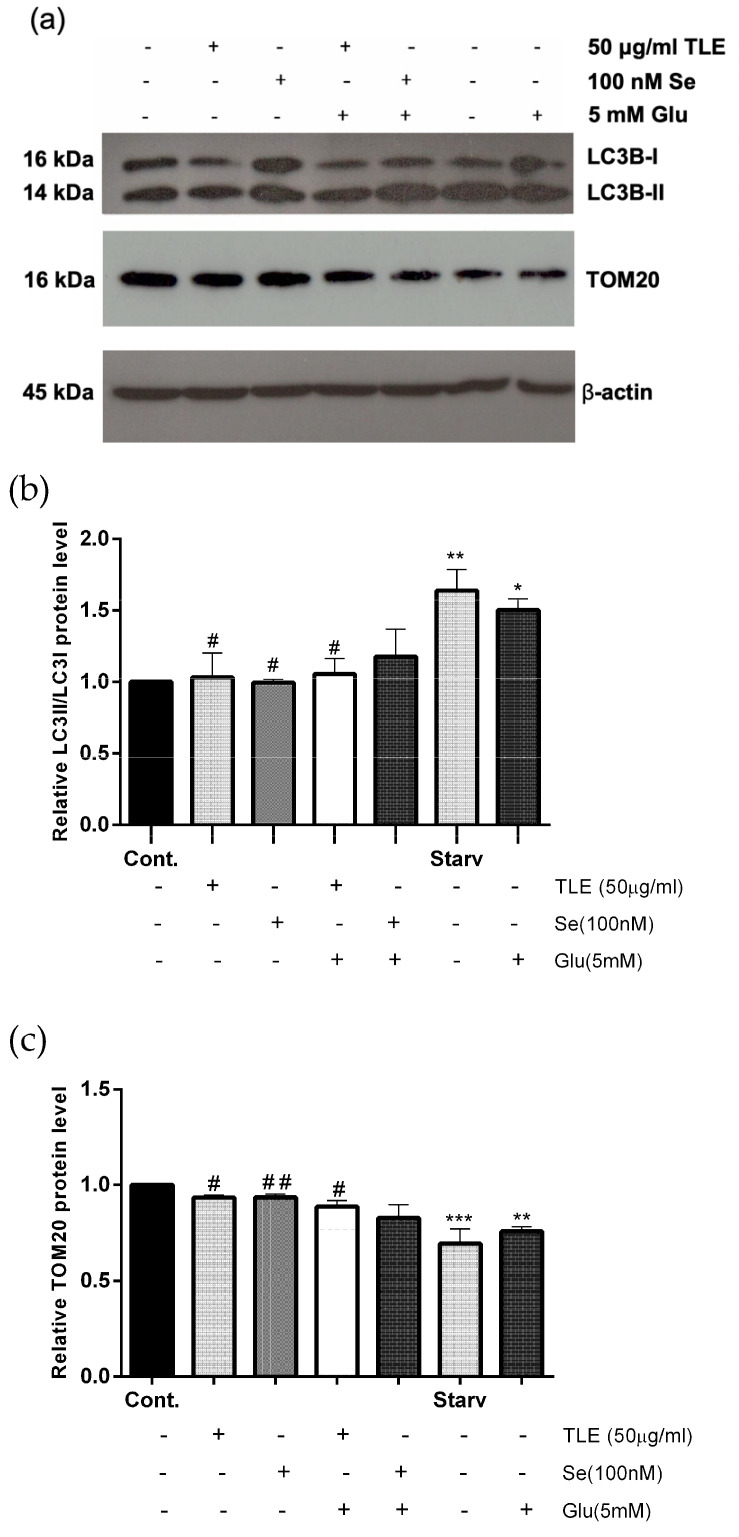
TLE inhibits glutamate-induced excessive mitophagy. HT-22 cells (passage 14,16,17) were pretreated with 50 µg/mL of TLE or 100 nM selenium followed by 5 mM glutamate for 18 h. (**a**) The protein expression level of LC3B (autophagy) and TOM20 (mitochondria) were analyzed by Western blot, and β-actin served as the loading control. Relative protein levels of (**b**) LC3B and (**c**) TOM20 were quantified by densitometry and the mean data from at least three independent experiments were normalized to the results. Cont, untreated control; Starv, starvation. The data represent the means ± SEM. * *p* value < 0.05, ** *p* value < 0.01, *** *p* value < 0.005 compared with untreated control ^#^ *p* value < 0.05, ^##^ *p* value < 0.01 compared with only the glutamate-treated group.

Prolonged generation of ROS and oxidative stress in neurons can promote autophagy and mitophagy processes [36,37,38]. Glutamate has been well known to induce oxidative stress and activate autophagy, leading to neuronal cell death in HT-22 cells [39,40]. To evaluate the correlation between glutamate-induced oxidative stress and an excessive mitophagy process, the protein expressions of specific autophagy and mitophagy markers, including the LC3 and mitochondrial protein (TOM20), were detected by Western blot analysis. LC3 is one of the most important proteins involved in the autophagy process, especially the LC3B isoform, and is often used as an autophagy marker. Moreover, the co-localization of LC3 and TOM20 are often used to represent the mitophagy process. Serum withdrawal (starvation), which is frequently used to increase the LC3-II level, was used as a positive control for autophagic flux [41]. HT-22 cells were pretreated with the most effective dose of TLE (50 µg/mL). Selenium (100 nM) was used as the positive control. We found that the treated cells with glutamate alone significantly increased the protein levels of the ratio of LC3B-II/LC3B-I, compared with the untreated group. Furthermore, both TLE and selenium treatment inhibited LC3B conversion compared with the untreated group (Figure 6a,b). In addition, the TOM20 protein expression level was significantly decreased in the glutamate treatment group compared with the untreated group, indicating that glutamate could cause the loss of mitochondrial protein. However, TLE and selenium treatment could sustain the TOM20 protein level compared with the untreated group (Figure 6a,c), indicating the maintenance of the mitochondrial protein by the extract upon glutamate treatment. These results indicate that TLE can inhibit glutamate-mediated oxidative stress and the excessive mitophagy in HT-22 cells.

**Figure 7:**
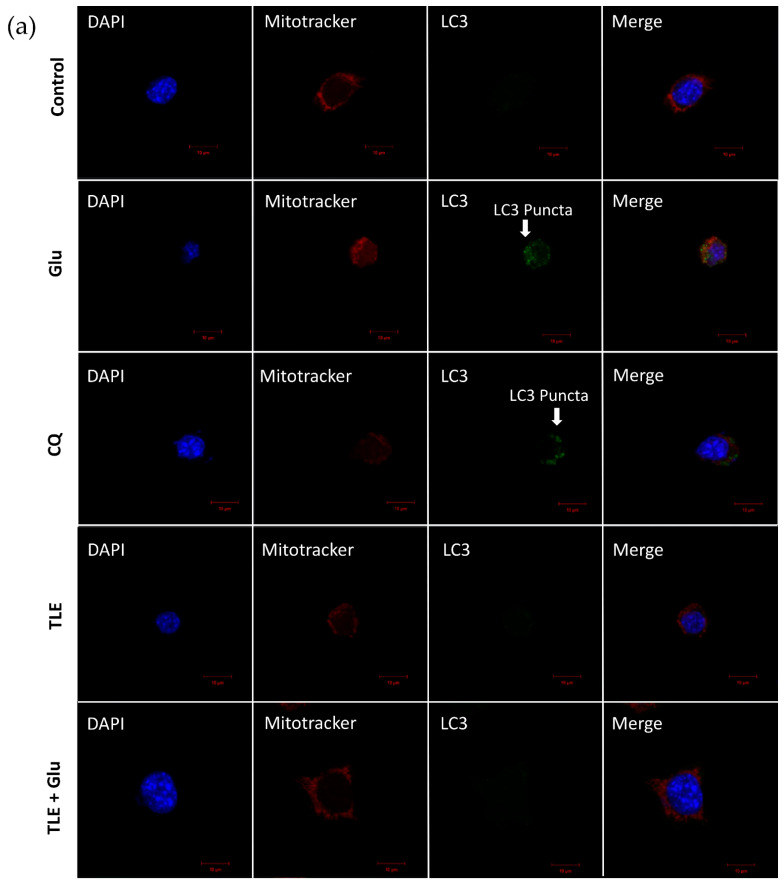

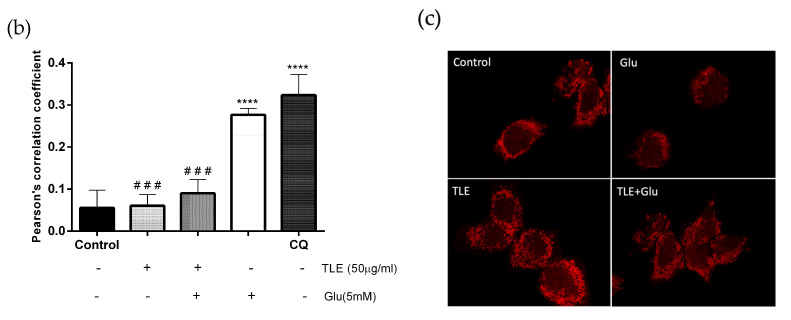

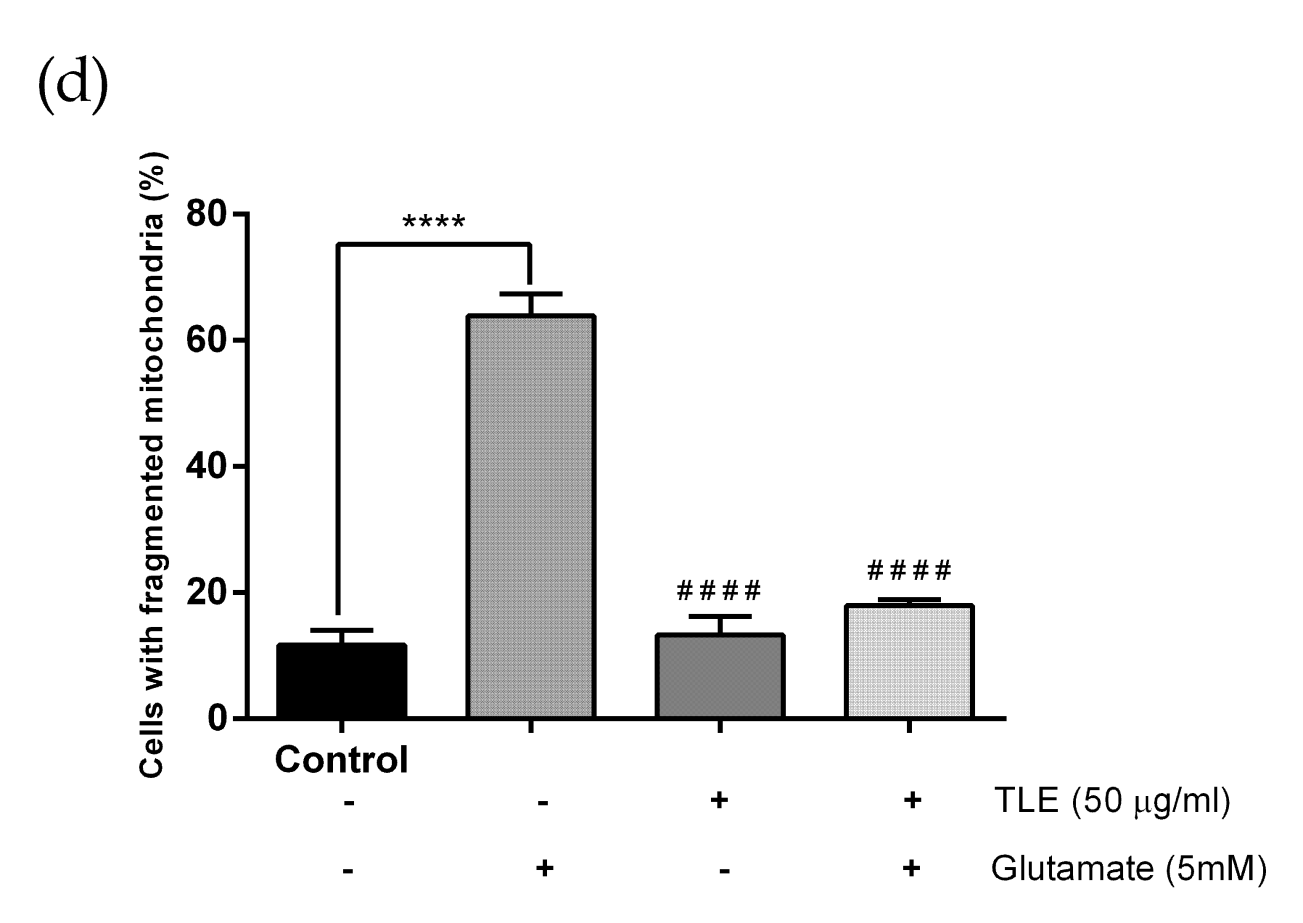
The immunofluorescence staining of LC3B protein and mitochondria. HT-22 cells (passage 10–12) were pretreated with 50 µg/mL of TLE followed by 5 mM glutamate for 18 h and 50 µM chloroquine (CQ) alone for 18 h is used as positive control (**a**) HT-22 cells were stained with the mitochondria (Mitotracker: red), LC3B protein (Alexa 488: green) and nucleus (DAPI: blue). They were observed under the confocal laser scanning microscope (scalebar is 10 µm). (**b**) The bar graph of co-localization was considered with Pearson’s correlation coefficient. (**c**) Micrograph of mitochondrial morphology. The normal morphology of mitochondria (tubular and round forms) was shown in the cell control. Glutamate altered the mitochondrial morphology, causing the mitochondrial fragmentation. (**d**) The numbers of cells with mitochondrial fragmentation were quantified as a percentage, data represent the means ± SEM (*n* = 3) and the averages of cells are at least 20 cells. **** *p* value < 0.001 compared with untreated control ^###^ *p* value < 0.005, ^####^ *p* value < 0.001 compared with glutamate-treated group.

To further clarify the glutamate-stimulated process of excessive mitophagy in HT-22 cells, the co-localizations of LC3B and mitochondria were assessed by immunocytochemistry assay to substantiate the protein expression results. LC3B was observed as punctate staining representing the autophagosomes. In this experiment, HT-22 cells were pretreated with 50 µg/mL of TLE and 100 nM selenium (positive control) for 24 h followed by 5 mM glutamate for 18 h. Moreover, chloroquine (lysosome inhibitor) was used as a positive control for autophagic flux [41]. Figure 7a exhibits that the control group showed no LC3 puncta formation, whereas glutamate treatment promoted the LC3 puncta formation, which was similar to that of 50 µM chloroquine (CQ) treatment (the autophagy control group). Noticeably, the pretreatment cells with TLE showed no LC3 puncta staining to cells, indicating the inhibition of autophagy (Figure 7a). Additionally, Pearson’s correlation coefficient was calculated. The co-localization occurred in the glutamate treatment group and autophagy control group (CQ), and was significantly suppressed by TLE treatment (Figure 7b). To demonstrate if the glutamate induced mitochondria dysfunction, the mitochondrial morphology was investigated (Figure 7c). Glutamate treatment led to mitochondrial fragmentation and also significantly increased the number of cells with fragmented mitochondria (Figure 7d). However, pre-treatment of 50 µg/mL TLE reduced the number of cells with fragmented mitochondria and prevented the glutamate-induced mitochondrial fragmentation, showing a normal morphology of mitochondria (tubular and round forms), the same as the cell control. Thus, our results indicate that glutamate could induce the overaccumulation of ROS, to further activate the excessive mitophagy process, leading to neuronal cell death. Taken together, these findings suggest that TLE provides neuroprotection by inhibiting the mitophagy signal.

### 3.7. In Silico Virtual Screening of Binding Affinity between TLE-Identified Compounds and Mitophagy Protein Markers

#### 3.7.1. Interaction between TLE-Identified Compounds and PINK1

**Figure 8:**
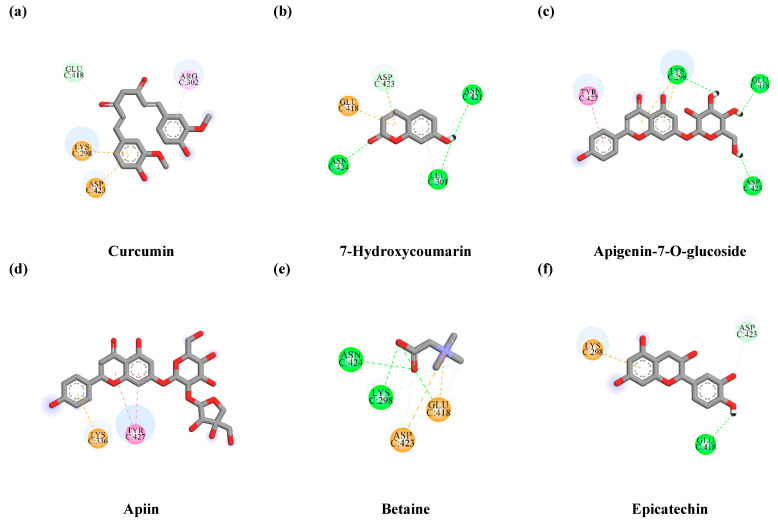
Schematics of amino acid interactions of PINK1 and candidate ligands: (**a**) curcumin, (**b**) 7-hydroxycoumarin, (**c**) apigenin-7-*O*-glucoside, (**d**) apiin, (**e**) betaine and (**f**) epicatechin. Green, pink and orange dashes indicate hydrogen, hydrophobic and electrostatic bonds, respectively.

The possible inhibitory effect of TLE-identified compounds on PINK1 activity was predicted by molecular docking study. Curcumin, a known natural PINK1 inhibitor [29], was utilized as a reference ligand in this study. Molecular docking results demonstrated that curcumin was docked into the binding pocket and interacted with LYS298 and LYS298 residues, similar to the previous report [29] (Table 3 and Figure 8). The binding affinity of curcumin at the PINK1 binding pocket was -5.4 kcal/mol. None of the candidate compounds had binding energies lower than the binding energy of curcumin. However, among all the candidate compounds, apigenin-7-*O*-glucoside showed the lowest binding affinity at -5.1 kcal/mol, which was closer to the affinity of curcumin (the reference ligand). The protein–ligand interactions are illustrated in Figure 8. These results imply that apigenin-7-*O*-glucoside was a candidate compound from TLE that suppressed the mitophagy process by inhibiting PINK1 activity.

#### 3.7.2. Interaction between TLE-Identified Compounds and E3 Ubiquitin-Protein Ligase Parkin

**Figure 9:**
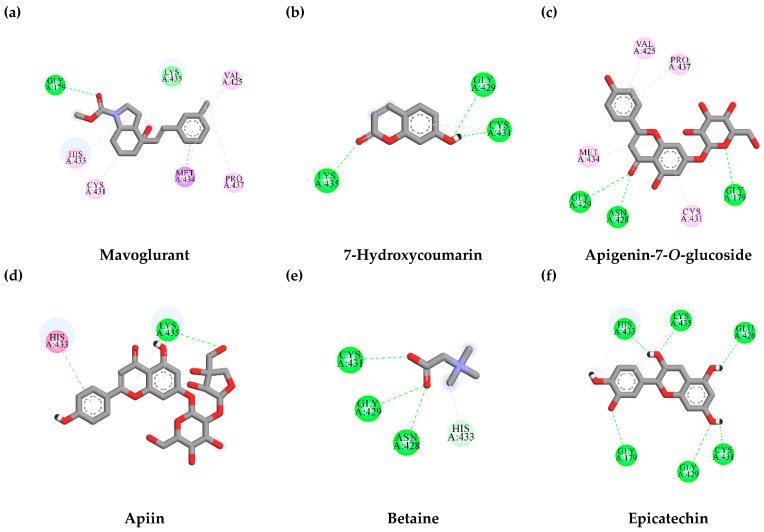
Schematics of amino acid interactions of E3 ubiquitin-protein ligase parkin and candidate ligands: (**a**) mavoglurant, (**b**) 7-hydroxycoumarin, (**c**) apigenin-7-*O*-glucoside, (**d**) apiin, (**e**) betaine and (**f**) epicatechin. Green, pink and orange dashes indicate hydrogen, hydrophobic and electrostatic bonds, respectively.

Parkin has a functional role in E3 ubiquitin-protein ligase and mediates the mitophagy process [42]. The targeting of parkin could provide therapeutic effects on neurodegenerative disorders [43]. In comparison, we studied the binding affinity of mavoglurant, an anti-Parkinson drug [44], which was reported to inhibit parkin activity [43]. The molecular docking results show that mavoglurant capably docked into the catalytic domain of parkin with a binding energy of −5.4 kcal/mol. Moreover, mavoglurant formed hydrophobic bonds with CYS431 and HIS433 residues, which were the conserved domain of E3 ubiquitin-protein ligase [45]. Interestingly, two out of five phytochemical compounds, namely epicatechin (−6.6 kcal/mol) and apigenin-7-*O*-glucoside (−6.2 kcal/mol), exhibited better binding affinities than mavoglurant (the reference ligand). The interactions between candidate compounds and E3 ubiquitin-protein ligase parkin at the catalytic site are shown in Table 4 and Figure 9. These results suggest that epicatechin and apigenin-7-*O*-glucoside could be potential candidate compounds from TLE that inhibit mitophagy by preventing E3 ubiquitin-protein ligase parkin activity.

### 3.8. Lipinski’s Rule of Five Parameters and ADMET Properties of TLE Phytochemical Compounds

The drug-likeness property of TLE’s phytochemical constituents was evaluated by Lipinski’s rule of five with the following criteria: molecular weight ≤ 500; the number of hydrogen bond acceptors ≤ 10; the number of hydrogen bond acceptors ≤ 5; and MlogP ≤ 4.15 [31]. Compounds that provided no more than one violation were considered drug-like compounds. As shown in Table 5, all TLE-identified compounds passed Lipinski’s criteria except apiin, which had an excess acceptable molecular weight and several hydrogen bond acceptors. In addition, pharmacokinetic properties, adsorption, distribution, metabolism, excretion, and toxicity (ADMET), of the identified compounds were predicted and are tabulated in Table 6. Overall, all compounds could absorb through the intestine. Interestingly, betaine and 7-hydroxycoumarin showed high intestinal absorption, and the percentage of absorption was 100% and 94.551%, respectively. Moreover, all identified compounds had no AMES toxicity and hepatotoxicity, except 7-hydroxycoumarin, which exhibited hepatotoxicity.

## 4. Discussion

Oxidative stress is one of the major causes of neurodegenerative disease, including AD [46,47]. It promotes the formation of neurofibrillary tangles, resulting in the progression of the disease [48,49,50]. Various studies showed that high glutamate content in the brain encourages ROS generation and leads to neuronal cell death, which is a pathophysiology of AD [10,51,52]. Some studies revealed that prolonged oxidative stress is associated with the mitophagy and autophagy processes, contributing to autophagic cell death in the neuronal system [19,53]. This process is also a significant regulator of other types of cell death, such as apoptosis and necrosis. Hence, the inhibition of glutamate-induced oxidative stress and neuronal cell death through mitophagy and autophagy processes may have the potential to provide a beneficial therapeutic approach for the treatment of neurodegenerative diseases.

In this study, we identified the phytochemical constituents of TLE, which was analyzed by LC–MS. The mass spectrum of LC–MS showed that TLE contains five major bioactive compounds (epicatechin, apigenin-7-*O*-glucoside, 7-hydroxycoumarin, apiin and betaine). Our data show that epicatechin is the most abundant bioactive compound in TLE, which could directly inhibit ROS and increase the gene expression of antioxidant enzymes [54,55]. From previous reports, it can be identified that epicatechin exhibits neuroprotection from traumatic brain injury and NMDAR-induced excitotoxicity through the Nrf-2-dependent antioxidant mechanism in animal models [56,57]. In addition, epicatechin ameliorates glutamate-induced oxidative and endoplasmic reticulum stress, and disrupts mitochondrial membrane potential by methamphetamine in HT-22 cells through the MAPK pathway [58]. Moreover, epicatechin was reported to have anti-inflammation, anticancer and neuroprotective properties [59,60]. Secondly, the water-soluble apigenin-7-*O*-glucoside (apigetrin) also has reported neuroprotective, antioxidant, anticancer, and antifungal properties [61,62,63,64]. Furthermore, 7-hydroxycoumarin (umbelliferone), a coumarin derivative, was also found in TLE. It has been reported to possess antioxidant activity by increasing the gene expression of antioxidant enzymes (SOD and CAT) [65], as well as antimicrobial and anticancer effects [66,67]. Umbelliferone also showed protection against glutamate toxicity due to its antioxidant potential [68]. Apiin (a glycoside of apigenin) and betaine were reported to have antioxidant potential [69,70,71]. Betaine exhibited neuroprotective activity against glutamate in primary cultured brain cells, which could be due to the stabilization of cell membranes from toxic insults or through the antioxidant potential [72]. For the neuroprotective approach, the effects of TLE against glutamate-induced oxidative toxicity in HT-22 cells and its underlying mitophagy mechanisms were investigated. In our present study, we found that TLE was able to increase cell viability and reduce ROS accumulation. Normally, ROS can be neutralized and scavenged through the antioxidant mechanisms of antioxidant enzymes such as SOD, GPx and CAT. Therefore, we analyzed the gene expression level of SOD1, SOD2, GPx and CAT. The results show that TLE can upregulate the expression of antioxidant enzymes. Interestingly, SOD2 regulation was higher when compared to other enzymes. SOD2 is located in the mitochondrial matrix and plays a significant role in the antioxidant process of mitochondria. Previous reports suggest that SOD2 enzyme reduces amyloid beta accumulation in Alzheimer’s mice [73,74]. Moreover, a decrease in SOD2 levels can cause the accumulation of Aβ protein [75]. Our study shows that TLE has potent antioxidant activity, and LC–MS analysis found that TLE had a high content of apigenin-7-*O*-glucoside (apigetrin), which is a well-known apigenin derivative [61]. Interestingly, Lim et al. (2016) reported that apigetrin enhances the expression of Nrf2 in HT-22 cells [63]. The presence of apigetrin in TLE could be responsible for promoting the antioxidant activity through Nrf2/ARE pathway. Thus, the increase in antioxidant enzyme genes links to the Nrf2/ARE-dependent signaling. Numerous lines of evidence indicate that Nrf2/ARE signaling can increase the gene expression level of various antioxidant enzyme genes in neurons [76,77,78,79]. Additionally, in order to predict the association between bioactive compounds of TLE and Nrf2 signaling, we also analyzed the in silico virtual screening of affinity between major bioactive compounds of TLE and KEAP1. It is a negative regulator of Nrf2. Interestingly, apigenin-7-*O*-glucoside possibly inhibited KEAP1, with a binding energy lower than that found in the reference ligand (GX8). 

Neuronal cells normally require the function of mitochondria as the main energy source. In order to study the effect of glutamate on mitochondrial status, TMRE (a fluorescent dye) was used to stain the active mitochondria. We found that glutamate can cause the loss of mitochondrial membrane potential, and TLE can restore the mitochondrial membrane potential status. This effect also involved the elevation of the endogenous antioxidant enzymes, namely, SOD1 and SOD2, CAT and GPx. Our study showed that glutamate treatment can produce the intracellular ROS and disrupt the mitochondrial membrane potential status in HT-22 cell model and can be reversed by TLE treatment. The findings indicate that TLE shows neuroprotective properties against glutamate-induced oxidative stress by directly suppressing the intracellular ROS generation, upregulating the antioxidant enzyme gene expression, and improving the mitochondrial membrane potential.

Mitophagy is a selective autophagy type, which is the most important process to eliminate the damaged mitochondria by recognizing the specific receptor, including PTEN-induced kinase1 (PINK1) and parkin. The mitophagy process is related to the oxidative stress conditions and central neurodegenerative diseases such as AD, PD, and ALS, etc. Especially in Parkinson’s patients, abnormality of PINK1 and parkin proteins leads to the disruption and accumulation of damaged mitochondria, and promotes oxidative stress in the nervous system [45,80]. However, the continuous stimulation of the mitophagy process results in autophagy-mediated cell death or stimulates cell death through another pathway. Interestingly, glutamate was found to induce neuronal cell death via the autophagy process, which occurs during prolonged oxidative stress, leading to excessive autophagy in neurons [36]. Moreover, the oxidative stress from starvation was also found to stimulate the autophagy-mediated cell death, along with ROS accumulation [81,82]. A recent study suggested that autophagy-mediated mitochondrial homeostasis plays an essential role in oxidative stress-linked neuronal damage and repair. Consequently, we investigated the specific protein markers (LC3B-I, LC3B –II, and TOM20) of the autophagy and mitophagy signaling pathway in response to glutamate treatment. Our results show that the treated cells with glutamate alone significantly activated the autophagy process by inducing the LC3B conversion ratio. However, the pre-treatment of the cell with TLE decreased the LC3-autophagic protein and restored the autophagy status. According to Kim et al., (2009), glutamate induced autophagy and caused neuronal cell death, which was further rescued by 3-methyladinine (3-MA) treatment [83]. The study suggests that autophagy activation is a key driver of neuronal cell death in response to glutamate in HT-22 mouse hippocampal cells. Our results indicate that TLE could inhibit glutamate-induced autophagy, resulting in the alleviation of neuronal cell death. Glutamate induces ROS accumulation, leading to mitochondrial disruption and a change in the morphology (fragmented) of mitochondria [34], which is in line with our study (Figure 7c,d). Thus, the damaged mitochondria are often the target of the autophagosome. A recent study showed that the high glutamate levels within neuronal cells result in increased expression of PINK1 and parkin proteins [84], which further play a significant role in the mitophagy signal. In addition, our molecular docking prediction of TLE showed the inhibitory effect on PINK1 and parkin. These results demonstrate that apigenin-7-*O*-glucoside exhibited better binding affinities against parkin than mavoglurant (the reference ligand) (Table 4 and Figure 9) and showed the lowest binding affinity against PINK1 at −5.1 kcal/mol, which was closer to the affinity of curcumin as the reference ligand (Table 3 and Figure 8). Thus, TLE may have the potential to inhibit mitophagy activation through PINK1 and parkin interaction. Mitophagy can be tracked by the measurement of LC3 protein expression in combination with mitochondrial-specific proteins, such as TOM20 and TIM23 [41,85]. Therefore, to investigate the association between ROS and mitophagy, we measured the protein expression of TOM20. Our results show that glutamate can cause the decrease in TOM20 protein expression level, which may be the result of the mitophagy process by the action of PINK1 protein, which plays an important role in the selective removal of damaged mitochondria. The damaged mitochondria can elevate PINK1 protein expression and recruit the parkin protein. These complexes trigger the initiation of mitophagy. Subsequently, mitochondrial proteins such as mitofusin, TOM20 and voltage-dependent anion channel (VDAC) are tagged with ubiquitin and become the target of p62 and LC3-II to enter the lysosome degradation process [48]. On the other hand, we revealed that TLE can increase the expression of TOM20 protein and inhibit autophagy activation. This may be due to the combination of increased mitochondrial biogenesis and the reduced mitochondrial damage through the cellular antioxidant Nrf2/ARE pathway. Nrf2 not only increases the expression of antioxidant enzymes, but is also involved in mitochondrial formation. It can activate nuclear respiratory factor 1 (NRF1), which regulates genes involved in mitochondria biogenesis, including mitochondrial transcription factor A (TFAM), mitochondrial transcription factor B1 (TFB1M) and mitochondrial transcription factor B2 (TFB2M) [86,87]. Furthermore, mitophagy can also be observed under confocal microscopy by tracking the co-localization of proteins in the autophagy process with mitochondrial-specific proteins. For example, the study on transgenic mice with abnormal cytochrome-c-oxidase (COX) resulted in the loss of energy production and promoted ROS accumulation along with co-localization of LC3 protein with mitochondria [88]. Similar results were also observed upon glutamate treatment. Interestingly, TLE was able to reduce the occurrence of LC3 puncta and co-localization in HT-22 cells, which is consistent with the results of LC3B and TOM20 protein expression. 

Furthermore, to elucidate and understand the pharmacokinetics properties, the ADMET analysis showed that all TLE-identified compounds can be absorbed, distributed, metabolized and excreted in the human body, along with low toxicity. Thus, TLE and its constituents with high BBB permeability may be appropriate for the study of neuroprotection. However, further studies to clarify the state of in vivo factors such as oral bioavailability rate and toxicity are required. 

## 5. Conclusions

**Figure 10:**
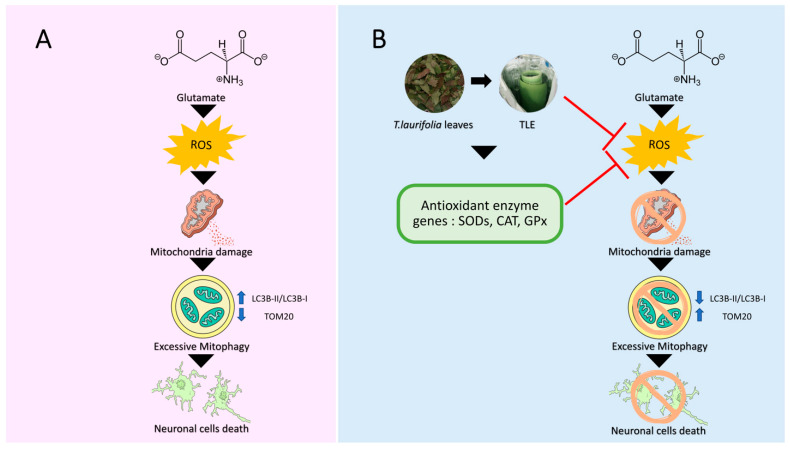
The summarized diagram of the neuroprotective effect and mechanism of TLE against glutamate-induced neuronal death in HT-22 cells through cellular antioxidants and mitophagy signaling. (**A**) The accumulation of glutamate in neurons leads to the rise in intracellular ROS. The high ROS can destruct the mitochondria and stimulate the excessive selective autophagy of damaged mitochondria (mitophagy), resulting in neuronal cell death. On the other hand, (**B**) TLE can directly neutralize the ROS and upregulate the gene expression of antioxidant enzymes. These two mechanisms completely inhibit ROS activity and rescue the mitochondrial health. Moreover, TLE can decrease the mitophagy activation, causing a decrease in neuronal cell death.

Excessive glutamate can increase the intracellular ROS accumulation in HT-22 cells and promotes neuronal cell death by inducing the loss of mitochondrial membrane potential, leading to excessive mitophagy-mediated cell death. Remarkably, TLE can reduce intracellular ROS accumulation by upregulating the expression of endogenous antioxidant enzyme genes in HT-22 cells. Consequently, a reduction in ROS accumulation maintains the mitochondrial membrane potential status and inhibits the excessive mitophagy-mediated neuronal cell death. Our findings provide the neuroprotective effect and mechanism of TLE in HT-22 cells through cellular antioxidants and mitophagy signaling. A summarized diagram of our findings is presented in Figure 10. Further studies are needed to clarify the underlying mechanism of TLE on Nrf2 signaling and mitophagy/autophagy. The data obtained from this study might potentially aid the development of alternative drugs to prevent or recover neurodegenerative disorders. 

## Figures and Tables

**Table 1:** List of primers.

Genes	Gene Accession Number	Sequence of Primer
SOD1 forward	NM_011434	5′-CAGGACCTCATTTTAATCCTCAC-3′
SOD1 reverse	NM_011434	5′-CCCAGGTCTCCAACATGC-3′
SOD2 forward	NM_013671	5′-CTGGACAAACCTGAGCCCTA-3′
SOD2 reverse	NM_013671	5′-TGATAGCCTCCAGCAACTCTC-3′
CAT forward	NM_009804	5′-CAGCGACCAGATGAAGCA-3′
CAT reverse	NM_009804	5′-CTCCGGTGGTCAGGACAT-3′
GPx forward	NM_008160	5′-ACAGTCCACCGTGTATGCCTTC-3′
GPx reverse	NM_008160	5′-CTCTTCATTCTTGCCATTCTCCTG-3′
β-actin forward	NM_007393	5′-GGCTGTATTCCCCTCCATCG-3′
β-actin reverse	NM_007393	5′-CCAGTTGGTAACAATGCCATGT-3′

**Table 2:** Molecular docking results of candidate ligands at the binding site of KEAP1 (PDB ID: 6HWS).

Ligand	Binding Energy (kcal/mol)	Amino Acid Interaction		
		Hydrogen Bond	Hydrophobic Bond	Electrostatic Bond
GX8 (reference ligand)	−8.6	SER363 ARG380 ASN414 ARG415 ARG483 (2) SER508 (2) SER555 SER602	TYR334 ALA556 TYR572	ARG415 (2) ARG483
7-Hydroxycoumarin	−6.5	SER363 ARG380 SER602	TYR334 (2) ALA556	-
Apigenin-7-*O*-glucoside	−8.7	SER363 ARG380 (2) ASN382 SER602	PHE478	ARG415 (2)
Apiin	−8.4	TYR334 SER363 (2) ARG380 ASN382 ASN414 SER508 SER555 SER602	ALA556	ARG415 (2)
Betaine	−3.9	ARG415 (3) SER508 (2) SER555	TYR525	TYR525
Epicatechin	−7.8	SER363 SER555 (2)	TYR334 TYR525 ALA556 TYR572	-

**Table 3:** Molecular docking results of candidate ligands at the binding site of PINK1 (PDB ID: 6EQI).

Ligand	Binding Energy (kcal/mol)	Amino Acid Interaction		
		Hydrogen Bond	Hydrophobic Bond	Electrostatic Bond
Curcumin (reference ligand)	−5.4	LYS298 GLU418	ARG302	LYS298 ASP423
7-Hydroxycoumarin	−4.9	LEU301 ASN421 (2) ASP423 ASN424	LEU301	GLU418 ASP423
Apigenin-7-*O*-glucoside	−5.1	LYS298 ASP423 GLU418	TYR427	LYS298 (2)
Apiin	−4.4	-	TYR427 (2)	LYS336
Betaine	−3.4	LYS298 (2) GLU418 (3) ASP423 ASN424	-	GLU418 ASP423
Epicatechin	−4.6	GLU418 ASP423	-	LYS298

**Table 4:** Molecular docking results of candidate ligands at the catalytic domain of E3 ubiquitin-protein ligase parkin (PDB ID: 5C1Z).

Ligand	Binding Energy (kcal/mol)	Amino Acid Interaction		
		Hydrogen Bond	Hydrophobic Bond	Electrostatic Bond
Mavoglurant (reference ligand)	−5.4	GLY179	VAL425 (2) CYS431 HIS433 MET434 (2) PRO437	-
7-Hydroxycoumarin	−4.8	GLY429 CYS431 LYS435	CYS431	-
Apigenin-7-*O*-glucoside	−6.2	GLY179 ASN428 GLY429	VAL425 CYS431 MET434 PRO437	-
Apiin	1.6	LYS435 (2)	HIS433 LYS435 (2)	-
Betaine	−3.4	ASN428 GLY429 CYS431 HIS433 (2)	-	-
Epicatechin	−6.6	GLY179 GLU426 GLY429 CYS431 HIS433 (2) LYS435	-	LYS435

**Table 5:** Physicochemical properties of TLE phytochemical compounds based on Lipinski’s rule of five parameters.

Compound	Molecular Weight (≤500)	#H-Bond Acceptors (≤10)	#H-Bond Donors (≤5)	MLOGP (≤4.15)	Lipinski #Violations (≤1)
7-Hydroxycoumarin	162.14	3	1	1.04	0
Apigenin-7-*O*-glucoside	432.38	10	6	−1.61	1
Apiin	564.49	14	8	−3.16	3
Betaine	117.15	2	0	−3.67	0
Epicatechin	290.27	6	5	0.24	0

# indicates number.

**Table 6:** ADMET properties of TLE-phytochemical compounds.

Pharmacokinetic Property	7-Hydroxycoumarin	Apigenin-7-*O*-Glucoside	Apiin	Betaine	Epicatechin
Absorption					
Water solubility (log mol/L)	−2.131	−2.559	−2.851	0.723	−3.117
Caco2 permeability (log Papp in 10-6 cm/s)	1.206	0.33	−0.966	1.44	−0.283
Intestinal absorption (human) (% Absorbed)	94.551	37.609	17.411	100	68.829
Skin permeability (log Kp)	−2.6	−2.735	−2.735	−2.78	−2.735
P-glycoprotein substrate	No	Yes	Yes	Yes	Yes
P-glycoprotein I inhibitor	No	No	No	No	No
P-glycoprotein II inhibitor	No	No	No	No	No
Distribution					
VDss (human) (log L/kg)	0.032	0.342	1.004	−0.304	1.027
Fraction unbound (human) (Fu)	0.432	0.218	0.171	0.875	0.235
BBB permeability (log BB)	−0.278	−1.391	−1.793	−0.214	−1.054
CNS permeability (log PS)	−2.741	−3.746	−4.972	−2.804	−3.298
Metabolism					
CYP2D6 substrate	No	No	No	No	No
CYP3A4 substrate	No	No	No	No	No
CYP1A2 inhibitior	Yes	No	No	No	No
CYP2C19 inhibitior	No	No	No	No	No
CYP2C9 inhibitior	No	No	No	No	No
CYP2D6 inhibitior	No	No	No	No	No
CYP3A4 inhibitior	No	No	No	No	No
Excretion					
Total Clearance (log ml/min/kg)	0.706	0.547	−0.054	0.326	0.183
Renal OCT2 substrate	No	No	No	No	No
Toxicity					
AMES toxicity	No	No	No	No	No
Max. tolerated dose (human) (log mg/kg/day)	0.689	0.515	0.446	0.838	0.438
hERG I inhibitor	No	No	No	No	No
hERG II inhibitor	No	No	Yes	No	No
Oral rat acute toxicity (LD_50_) (mol/kg)	2.047	2.595	2.49	1.654	2.428
Oral rat chronic toxicity (LOAEL) (log mg/kg_bw/day)	1.751	4.359	4.574	0.254	2.5
Hepatotoxicity	Yes	No	No	No	No
Skin sensitization	No	No	No	Yes	No
*T.Pyriformis* toxicity (log ug/L)	0.546	0.285	0.285	−0.057	0.347
Minnow toxicity (log mM)	1.714	5.507	3.835	2.97	3.585

BBB: blood–brain barrier, BB: brain:blood drug concentration ratio, CNS: central nervous system, PS: permeability–surface area.

## Data Availability

Data is contained within the article and Supplementary Materials.

## Supplementary Materials

The following are available online at https://www.mdpi.com/article/10.3390/antiox10111678/s1, Figure S1: Analysis of bioactive compounds in ethanolic *Thunbergia laurifolia* leaf extract by liquid chromatography-mass spectrometry (LC-MS) technique.
