# Supplementary material for: Thunbergia laurifolia Leaf Extract Inhibits Glutamate-Induced Neurotoxicity and Cell Death through Mitophagy Signaling
Source: Antioxidants (Basel). 2021 Oct 25;10(11):1678. doi: 10.3390/antiox10111678 (PMC8614718; doi:10.3390/antiox10111678)

Figure S1 : Analysis of bioactive compounds in ethanolic Thunbergia laurifolia leaves extract by liquid chromatography-mass spectrometry (LC-MS) technique.

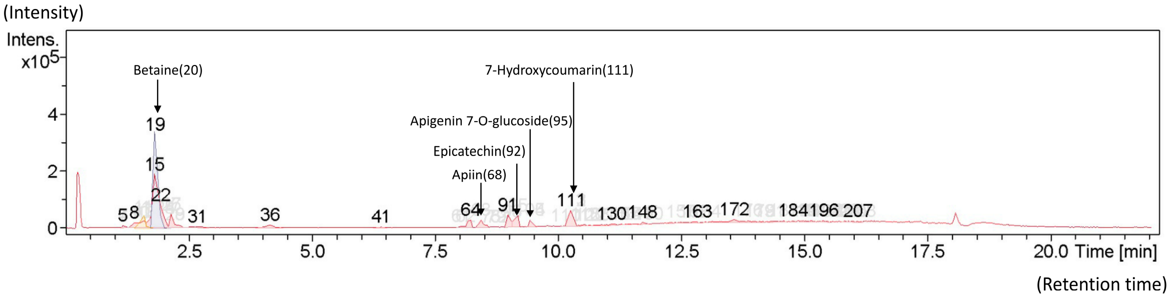

Supplement: Supplementary file 1 [file antioxidants-10-01678-s001.zip › antioxidants-1338251-supplementary.pdf]
